# Prevalence of Congenital Anomalies and Its Associated Factors Among Newborns in Central Ethiopia Region Public Hospitals, Ethiopia: A Retrospective Cross‐Sectional Study, 2023

**DOI:** 10.1002/puh2.70154

**Published:** 2025-10-30

**Authors:** Daniel Tsega, Atsede Getawey

**Affiliations:** ^1^ Department of Midwifery College of Medicine and Health Science Wolkite University Wolkite Ethiopia; ^2^ Department of Biomedical College of Medicine and Health Science Wolkite University Wolkite Ethiopia

**Keywords:** congenital anomalies, Ethiopia, functional defects, newborns, structural deformity

## Abstract

**Introduction:**

A congenital anomaly (CA) is an anatomical, physiological, and metabolic defect of newborns that might be recognized during pregnancy, delivery, or later in life. CAs are structural and/or functional defects that have a significant physical, metabolic, mental, and developmental health of the child, as well as affected social and cosmetic consequences for the child, and require surgery or medical treatment.

**Methods:**

An institution‐based retrospective cross‐sectional study was done among 3427 newborn mother registration charts. Data were collected using pretested, structured questionnaires by chart review, which was coded, checked, and entered into EpiData software version 4.6, then exported to Statistical Package for Social Science software version 25 for further analysis. Bivariable analysis was done to see the crude significant relation of each independent variable with the dependent variable. *p* values at <0.25 during the bivariable analysis were entered into multivariable analysis to see the net effect of confounding variables. Adjusted odds ratios (AORs) with 95% confidence intervals (CIs) were reported. Finally, the variable *p* value <0.05 was declared as statistically significant.

**Result:**

The overall birth prevalence of CAs was 27 per 1000 births. The most commonly observed anomalies included central nervous system, orofacial clefts, and Down syndrome, with prevalence of 13, 8, and 7 per 1000 births, respectively. Factors significantly associated with CAs were rural area reside (AOR: 5.2, 95% CI: 1.98–13.68), women with no formal education (AOR: 7.8, 95% CI: 2.93–20.98), history of CAs (AOR: 3.08, 95% CI: 1.28–7.38), exposure of pesticide (AOR: 8.9, 95% CI: 3.54–22.63), and lack of folic acid supplementation during pregnancy (AOR: 2.52, 95% CI: 1.17–5.43).

**Conclusion:**

CAs remain a substantial public health concern in the Central Ethiopia region. Strengthening antenatal care services, promoting periconceptional folic acid supplementation, and minimizing maternal exposure to environmental risk factors may reduce the prevalence.

## Graphical abstract

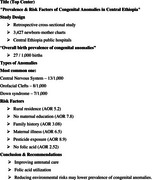

## 1 Introduction

Congenital anomalies (CAs) are structural, functional, and/or metabolic abnormalities that occur during intrauterine life and can be identified during pregnancy, delivery, or later in life [1]. CAs often cause significant problems of medical, physical, mental, social, and cosmetic results for the affected child that require surgery or medical treatment [2, 3, 4]. During the first trimester of pregnancy, the stage of embryonic development, or embryogenesis, is especially susceptible to interruption by internal and external teratogenic factors that may lead to many CAs, which affect single or multiple systems and may lead to significant lifelong disability or mortality [5, 6].

The overall birth prevalence of congenital anomalies is the fifth leading cause of reduced life expectancy before reaching the age of 67.32 of point of life expectancy (average life expectancy of Ethiopian people) [7]; as there is a defect either in the structure or function of the body, the body system, and the immunity, it does not work as normal function. Due to this reason, the life expectancy is reduced from the normal living age of the country. Globally, around 295,000 babies are born with birth defects that die within their neonatal period every year due to CAs [1]. Each year 3.3 million under‐five children die related to birth defects as well as 3.2 million children who survive CAs lifelong physical and mental disability as well and 303,000 neonates die during their neonatal period due to birth defects [4, 8]. CAs severely affected 3% of live births and 20% of stillbirths [3, 9]. CAs are affected seriously in both developed and developing countries by neonate mortalities and disabilities, with 94% birth defects, 95% mortality, and 15%–30% of hospital admissions of neonates due to CAs in low‐ and middle‐income countries [10].

Globally, CAs affect approximately 3%–6% of infants and contribute to 240,000 neonatal deaths every year, specifically in low‐ and middle‐income countries where access to preventive and curative care is limited [7]. The prevalence of CAs varies from country to country; in Brazil, the incidence varies from 0.49% to 1.7% of all births [11, 12, 13], in Iran 2.3% [7], in Lebanon 2.4% [14], in China 3.58% [15], and in Saudi Arabia 5.9% [16]. The incidence of CAs in Africa was shown in Nigeria at 6.3% [17], in Egypt at 2.5% [18], and in Tanzania at 29%, which is the highest incidence of CAs in other countries [19]. The prevalence of CAs in Ethiopia ranged from 1.99% to 5.95% [20, 21, 22]. The risk factors of CA were included genetic factors, environmental factors, maternal medical diseases, substance abuse, and micronutrient deficiency. Especially, lack of folic acid supplementation before and during pregnancy has relations with the occurrences of CAs [23, 24, 25].

Minor CAs are minor structural abnormalities recognized at birth, which are the most common among the populations that have no significant health problem before surgery in the neonatal period and also have minor social or cosmetic consequences for the affected persons, whereas the major CAs categories have major anatomical and physiological abnormalities that are enough to reduce life expectancy and have a significant impact on body function that lead to stillbirth, difficulty of newborn survival, neonatal death, and a significant problem on cosmetics appearance [22, 26]. Anencephaly, hydrocephalous, spinal bifida, gastrointestinal defect, cleft lip, cleft palate, clubfoot, and Down syndrome are the most common major CAs identified during pregnancy, delivery, and later life of neonates [27].

The Sustainable Development Goals (SDGs) aim to reduce under‐five mortality to at least 25 per 1000 live births and newborn mortality to at least 12 per 1000 live births by the end of 2030 [28]. Addressing and preventing CAs is essential to achieve this SDG successfully as congenital birth defects are significant contributors to neonate mortality, accounting for 9% of neonate deaths [29].

In Ethiopia, including in Central Ethiopia, many babies are born with different CAs that affect their survival. The risk factors of CAs have not been well studied. Therefore, identifying the precise risk factors for congenital malformations is essential for delivering health education to raise awareness and for implementing prophylactic measures to develop strategic prevention plans. Though intervention strategies were being practiced, the burden of CAs remains high in Ethiopia. These burdens suggest that further study is needed on CAs. Limited evidence was available on the contributing factor of CAs, especially in the Central Ethiopia regions. As such, this study aims to measure the birth prevalence and associated factors of CAs among newborns in Central Ethiopia regional public hospitals from March 3, 2023, to April 30, 2023.

## 2 Methods and Materials

### 2.1 Study Design, Setting, and Population

A cross‐sectional study was conducted using medical records from registered newborn patients and medically terminated fetuses in Central Ethiopia region public hospitals. Central Ethiopia is one of the regions of Ethiopia. It is found southwest of Addis Ababa, the capital city of Ethiopia. There are seven administrative zones and two special districts. In the region, there are 25 public hospitals, which serve the total population in the region. All hospitals provide comprehensive emergency obstetric care services.

Gurage zone have five public hospitals: four primary and one specialized teaching hospital; two of them (one primary and the specialized teaching hospital) were selected; in the Kembata zone, there are five hospitals: four primary and one general hospital; three of them (two primary and the general hospital) were selected; and in Halaba zone, there are three hospitals; one general hospital was selected by lottery method for this study.

The study was conducted from March 3, 2023, to April 30, 2023, on charts that record from February 30, 2021, to February 30, 2023.

Newborns and medically terminated fetuses (gestational age >12 weeks medical registered chart) registered in one of the six numbers of regional public hospitals between February 30, 2021, and February 30, 2023, who were included in the study.

### 2.2 Inclusion Criteria

All newborns and medically terminated fetuses (gestational age >12 weeks medical registered chart) from February 30, 2021, to February 30, 2023, were included.

### 2.3 Exclusion Criteria

- Charts with no registration number were excluded.
- Records with incomplete information that miss two or more variables were excluded.
- Charts of medically terminated fetuses whose gestational age was less than 12 weeks were excluded.

### 2.4 Sample Size Determination

The sample size was determined by taking all newborn babies delivered in Central Ethiopia region public hospitals from February 30, 2021, to February 30, 2023, were included in the study. All 3427 newborns from February 30, 2021, to February 30, 2023 with medical registered charts were reviewed to identify newborn babies with CAs.

### 2.5 Data Quality Control and Procedure

After reviewing pertinent literature, pretested and organized questionnaires were developed as a data‐gathering tool. The data were collected through a review of charts. Six trained BSc midwives and one supervisor (MSc midwife) ascertained data from patient charts. The data collectors reviewed the charts from the selected hospitals. The tool consists of socio‐demographic factors, obstetric history, medical illnesses, and drug intake, exposure to radiation, CAs, history in the family, maternal exposure to environmental factors, genetic factors, neonate factors, and history of periconceptional folic acid supplementation.

To assure the data quality, training was given to the data collectors and supervisor for 2 days on the objective, relevance of the study, pretest, and ways of chart review. Before going to data collection, a pretest was done on 5% of the total sample size at one of the Gurage zone public hospitals that is not part of the study site to ensure the validity of the survey tool and to standardize the questionnaire. Each questionnaire was checked for completeness and missed values; those incomplete questionnaires were managed accordingly. To ensure the accuracy and consistency of the data reviewed daily, the primary investigator and supervisors were double‐check and evaluate completed questionnaires.

### 2.6 Sampling Technique and Procedure

The newborn mother's medical registered chart from February 30, 2021, to February 30, 2023, which fulfills inclusion criteria, was reviewed. The data were collected through a retrospective review of charts of all mothers who delivered at selected hospitals of live births, stillbirths, and medically terminated fetuses due to CAs. Their medical record numbers recorded on the admission log books in the record class were used as sample frames to review all the required information on the charts. A census method was applied to review the charts in selected hospitals, which means that a consecutive sampling method was employed to select the study participants until the desired sample size was achieved.

### 2.7 Operational and Term Definitions

**Congenital anomaly**: Any structural and/or functional abnormality of intrauterine origin identified before or after birth. So newborns who have one birth defect are considered CAs.
**Anencephaly**: It is a severe defect involving the absence of the entire brain or cerebral hemisphere, which results from the defect in the closure of the neural tube during fetal development.
**Spinal bifida**: This spine deformity, which can occur in any spine but is more prevalent in the lumbosacral areas, is caused by the posterior part of the vertebral column's lamina failing to close.
**Hydrocephalus**: It is a condition caused by abnormalities in the production, absorption, or flow of actual cerebrospinal fluid within the intracranial space, which is characterized by excessive accumulation of fluid in the brain.
**Down syndrome**: It is the most common chromosomal abnormality observed in humans due to an extra “chromosome 21,” which is characterized by physical and mental handicaps.
**Major anomaly**: A significant defect that affects viability or requires surgical/medical intervention (e.g., neural tube defects and cardiac anomalies).
**Minor anomaly**: A defect with minimal functional or cosmetic consequence, not typically requiring urgent intervention.
**Census method**: Inclusion of all eligible study subjects (charts) without applying a sampling technique.

### 2.8 Data Processing and Analysis

The collected data were checked visually for its completeness, and the collected information was coded and entered into EpiData version 4.6 statistical packages and then exported to SPSS version 25 for statistical analysis. Then the analyzed data were presented using text, tables, and graphs. Bivariable logistic regression was used to identify crude associations between each independent variable and CAs. Variables with *p* values < 0.25 in the bivariable analysis were entered into the multivariable logistic regression model to assess adjusted associations while controlling for potential confounders. The goodness of model fitness was tested by the Hosmer–Lemeshow statistical test which is insignificant. Adjusted odds ratios (AORs) with 95% confidence intervals (CIs) were calculated to measure the degree of association between independent and dependent variables. Finally, the variables with a *p* value less than 0.05 were considered statistically significant.

## 3 Results

### 3.1 Socio‐Demographic Characteristics

In total, 3427 newborns and terminated fetuses were included in the study. The mean ages of neonates and their mothers were 5.68 ± 3.09 days and 27.7 ± 4.76 years, respectively. Around two‐thirds of 2204 (64.3%) neonates were within 7 days of age, and 2954 (86.2%) neonate's mothers were between 20 and 34 years of age group. Of the total neonates included in this study, half of 1716 (50.1%) were male. More than half of 1954 (57.0%) neonate mothers were housewives. The majority of neonate mothers, 2889 (84.3%), were married (Table 1).

**TABLE 1:** Socio‐demographic characteristics of respondents among neonates born in Central Ethiopia region public hospitals, Ethiopia 2023 (*n* = 3427).

Variables	Category	Frequency	Percentage
Mother's age	<20 years	41	1.2
	20–34 years	2954	86.2
	≥35 years	432	12.6
Neonate's age	≤7 days	2204	64.3
	≥8 days	1223	35.7
Newborn status	Live births	3173	92.6
	Stillbirths	238	6.94
	Terminated fetus	16	0.46
Neonate's sex	Male	1716	50.1
	Female	1711	49.9
Birth order	First	1004	29.2
	Second	1111	32.4
	Third	604	17.6
	Fourth	350	10.2
	Fifth and above	358	10.4
Mother's religion	Orthodox	1389	40.5
	Protestant	1296	37.8
	Muslim	492	14.4
	Catholic	250	7.3
Mother's ethnicity	Gurage	2098	61.2
	Kembata	728	21.2
	Halaba	193	5.6
	Amhara	347	10.2
	Others	61	1.8
Occupational status of the mother	Housewife	1954	57.0
	Factory worker	12	0.4
	Employee	663	19.3
	Merchant	476	13.9
	Student	322	9.4
Educational status of the mothers	Non‐educated	651	19.0
	Primary education	1107	32.3
	Secondary and above	975	28.4
	Occupational training	9	0.3
	Diploma and above	685	20.0
Mother's marital status	Single	200	5.8
	Married	2889	84.3
	Divorced	240	7.0
	Widowed	98	2.9
Residence	Rural	1723	50.3
	Urban	1704	49.7

*Note:* Other of mother's ethnicities include Hadiya and Silte.

### 3.2 Maternal Genetical Factors

Among the reviewed charts, 60 (1.8%) had a family history of CAs. In addition, 93 (2.7%) of women had a history of CAs in previous gravidity/parity.

### 3.3 Maternal Reproductive and Obstetric History

Among the reviewed charts, around two‐thirds of 2030 (59.2%) women had 2–4 parity. About 311 (9.1%) of women had a history of abortion. One hundred fifty‐two (4.4%) of the neonate's mothers had a history of newborn death, and two‐thirds of them had early neonatal death. About 9.2% of neonate mothers had no ANC visit, and 52.1% of women had at least four ANC visits (Table 2).

**TABLE 2:** Reproductive and obstetric history of respondents among neonates born in Central Ethiopia region public hospitals, Ethiopia, 2023 (*n* = 3427).

Variables	Category	Frequency	Percentage
Number of parity	One	1062	31.0
	Two to four	2030	59.2
	Five and above	335	9.8
History of abortion	No	3116	90.9
	Yes	311	9.1
History of newborn death	No	3275	95.6
	Yes	152	4.4
Type of newborn death (*n* = 152)	Stillbirth	51	33.6
	Early neonatal death	101	66.4
Was pregnancy planned	No	537	15.7
	Yes	2890	84.3
Gestational age at first visit	No ANC visit	314	9.2
	1–3 month	1644	48.0
	4 and above months	1469	42.8
Number of ANC visit (*n* = 3113)	One	67	2.2
	Two	330	10.6
	Three	936	30.1
	Four and above	1780	57.2
Place of ANC visit (*n* = 3113)	Public health institution	3096	99.5
	Private health institution	17	0.5
Number of preterm delivery	No preterm birth	3370	98.3
	Yes	57	1.7

### 3.4 Maternal Medical and Drug History

Among the total of women's reviewed charts, 155 (4.5%) had a history of chronic diseases, and out of them, 137 (88.4%) were not treated. A total of 1656 (48.3%) of women were used the hormonal contraceptive method. About 220 (6.4%) of women had a history of febrile illness, and from those, 153 (69.5%) had used antipyretic drugs. Among the study subjects, 145 (4.2%) were exposed to pesticides during pregnancy (Table 3).

**TABLE 3:** Maternal medical history and chemical exposure during pregnancy among neonates born in Central Ethiopia region public hospitals, Ethiopia, 2023 (*n* = 3427).

Variables	Category	Frequency	Percentage
History of medical disease	No	3272	95.5
	Yes	155	4.5
Treatment (*n* = 155)	No	137	88.4
	Yes	18	11.6
Hormonal contraceptive	No	1771	51.7
	Yes	1656	48.3
Febrile illness	No	3207	93.6
	Yes	220	6.4
Antipyretic drug (*n* = 220)	No	67	30.5
	Yes	153	69.5
Pesticide	No	3282	95.8
	Yes	145	4.2
Women have used hot bath	No	3419	99.8
	Yes	8	0.2
Radiation	No	3369	98.3
	Yes	58	1.7

### 3.5 Maternal Nutritional and Folic Acid Consumption

The study participants had a mean BMI of 21.17 ± 2.47, with 2752 (80.3%) women falling within the normal range. Among the reviewed charts, 2467 (72.0%) of women obtained folic acid supplements after 3 months of conception during the first antenatal care follow‐up visit (Table 4).

**TABLE 4:** Maternal nutrition and folic acid consumption of respondents among neonates born in Central Ethiopia region public hospitals, Ethiopia, 2023 (*n* = 3427).

Variables	Category	Frequency	Percentage
Folic acid supplements	Never	655	19.1
	Preconception intake	305	8.9
	After 3 months of conception	2467	72.0
Categorized BMI	Underweight	446	13.0
	Normal	2752	80.3
	Overweight	220	8.4
	Obesity	9	0.3

### 3.6 Maternal Lifestyle

Around two‐thirds of 2074 (60.5%) women have taken caffeine. Among these coffee drinkers, the widely held 1666 (80.3%) women have taken three or more cups of coffee daily. Regarding alcohol consumption, 368 (10.7%) of women have used alcohol (Table 5).

**TABLE 5:** Maternal lifestyle among neonates born in Central Ethiopia region public hospitals, Ethiopia, 2023 (*n* = 3427).

Variables	Category	Frequency	Percentage
Taking caffeine	No	1353	39.5
	Yes	2074	60.5
Types of caffeine (*n* = 2082)	Coffee	2064	99.1
	Tea	18	0.9
Dose of coffee in a cup (*n* = 2074)	≤2 cup/day	408	19.7
	≥3 cup/day	1666	80.3
Alcohol drinking	No	3059	89.3
	Yes	368	10.7
Dose of alcohol (*n* = 368)	>500 mL/day	211	57.3
	≤500 mL/day	79	21.5
	˂500 mL/week	78	21.2
Smoking	No	3354	97.9
	Yes	73	2.1
Passive smoking	No	3372	98.4
	Yes	55	1.6

### 3.7 Prevalence of CAs Among Newborns

**FIGURE 1:**
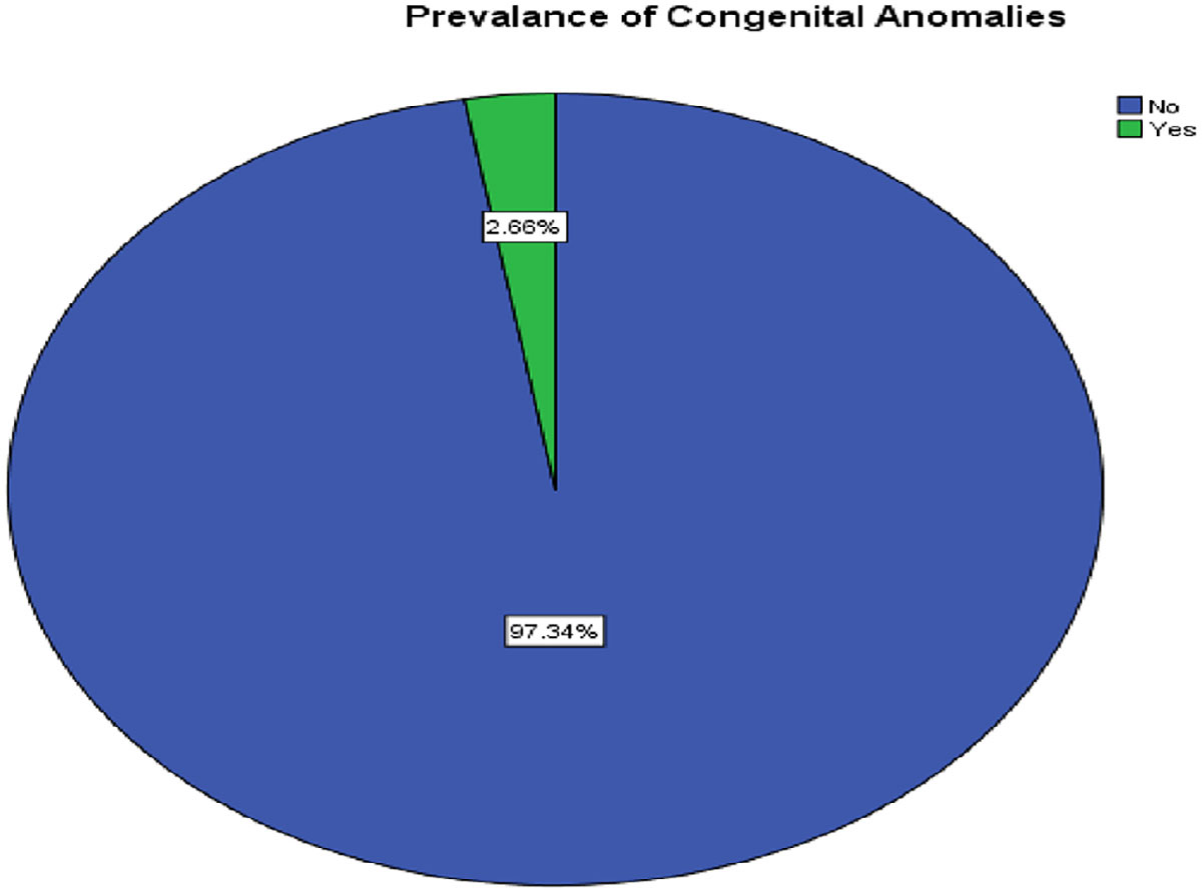
Prevalence of congenital anomalies among neonates born in Central Ethiopia region public hospitals, Ethiopia, 2023 (*n* = 3427).

The total prevalence of CAs in this study was 2.7% (95% CI: 2.1%–3.2%) (Figure 1).

### 3.8 Types of CAs

**FIGURE 2:**
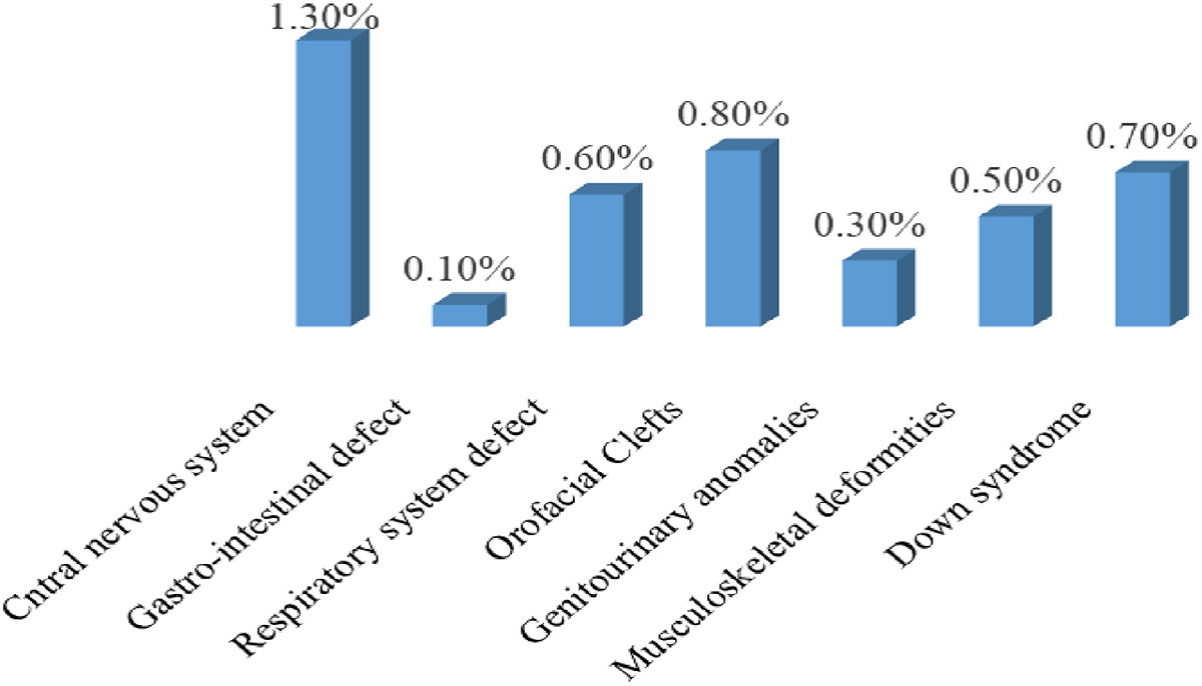
Distribution of types of congenital anomalies among women who gave birth in Central Ethiopia region public hospitals, Ethiopia, 2023 (*n* = 3427).

The most commonly identified types of CAs were central nervous system 43 (1.3%); among these, anencephaly 22 (0.6%) and spinal bifida 21 (0.6%) were the most commonly prevalent central nervous system defects, whereas genitourinary system 10 (0.3%) and gastrointestinal system 3 (0.1%) were less common identified types of CAs (Figure 2).

### 3.9 Factors Associated With CAs

In binary logistic regression, maternal age, residence, educational status, history of CA, abortion, use of any caffeine, febrile illness, pesticide exposure, use of any hormonal contraceptives, folic acid supplements, and newborn death were associated with CAs in bivariable analysis at *p* value ≤ 0.25. However, in multivariable analysis, maternal educational status, residence, history of CA, febrile illness, history of abortion, pesticide exposure, and folic acid supplementation were significantly associated with CAs at *p* value < 0.05.

Newborns of mothers who reside in rural areas were five times more likely to have CAs as compared to those who reside in urban (AOR: 5.2, 95% CI: 1.98–13.68). Newborns of mothers with no joined formal education were eight times more likely to have CAs than those who attended formal education (AOR: 7.8, 95% CI: 2.93–20.98). Newborns of mothers who had a history of CAs in previous birth were three times increases the newborns’ CA than counterparts (AOR: 3.08, 95% CI: 1.28–7.38). Women who had medical illnesses were six times more likely to deliver congenitally malformed offspring than their counterparts (AOR: 6.5, 95% CI: 3.07–13.77). Newborns of mothers who had a history of abortion were three times more likely to have CAs than their counterparts (AOR: 2.81, 95% CI: 1.40–5.64). Newborns of the mother who had exposure to pesticides were nine times more likely to deliver congenitally malformed offspring than the counterpart (AOR: 8.9, 95% CI: 3.54–22.63), and those who do not take folic acid supplements before and during pregnancy were three times more likely to deliver congenital malformed newborns than those counterparts (AOR: 2.52, 95% CI: 1.17–5.43) (Table 6).

**TABLE 6:** Binary logistic regression analysis to identify factors associated with newborn congenital anomalies in Central Ethiopia region public hospitals, Ethiopia 2023 (3427).

Variables	Category	Congenital anomaly				95% CI FOR AOR		
		No	Yes	COR (95% CI)	AOR	Lower	Upper	*p* value for AOR
Mother age	<35 years	2955	40	1	1			
	≥35 years	381	51	9.88 (6.45–15.16)	1.05	0.336	3.27	0.935
Residence	Rural	1641	81	8.37 (4.32–16.19)	5.20	1.98	13.68	0.001
	Urban	1695	10	1	1	0.592	8.192	0.238
Educational status	Illiterate	615	36	5.37 (3.03–9.53)	7.84	2.93	20.98	0.000
	Primary	1070	37	3.17 (1.80–5.60)	0.48	0.18	1.25	0.133
	Secondary and above	1651	18	1	1			
Women had Hx of CA	Yes	47	46	71.5 (43.31–118.14)	3.08	1.28	7.38	0.012
	No	3289	45	1	1			
Use of any caffeine	Yes	1998	76	3.39 (1.94–5.93)	0.67	0.319	1.383	0.274
	No	1338	15	1	1			
Medical illness	Yes	157	63	45.5 (28.38–73.12)	6.50	3.07	13.77	0.000
	No	3179	28	1	1			
History of abortion	Yes	274	37	7.66 (4.95–11.84)	2.81	1.40	5.64	0.004
	No	3062	54	1	1			
Pesticide exposure	Yes	84	61	78.7 (48.3–128.2)	8.95	3.54	22.63	0.000
	No	3252	30	1	1			
Newborn death	Yes	97	55	51.01 (32.0–81.3)	2.51	0.87	7.25	0.088
	No	3239	36	1	1			
Hormonal contraceptive	Yes	1576	80	8.12 (4.30–15.31)	1.97	0.87	4.47	0.104
	No	1760	11	1	1			
Folic acid supplements	Never received	585	70	15.67 (9.55–25.73)	2.52	1.17	5.43	0.018
	Received	2751	21	1	1			

Abbreviations: AOR, adjusted odds ratio; CA, congenital anomalies; CI, confidence interval.

## 4 Discussion

The overall prevalence of CAs was 2.7% (95% CI: 2.1%–3.2%). which is consistent with prior studies done in Egypt 2.5% [18], a study done in Iran 2.3% [7], a study done in eastern India 2.22% [30], and a study done in Lebanon 2.4% [14]. But lower than the study conducted in Jimma, Ethiopia 5.95% [22], the study conducted in Nigeria 6.3% [17], the study conducted in Mwanza, Tanzania 29% [19], and the study done in Afghanistan 7.5% [31]. The discrepancy might be due to the study setting, and the time variation might be due to the variation in the health care service accessibility and coverage in the study areas. This finding was higher than the study conducted in Addis Ababa and the Amhara region 1.99% [21], the study done in Bahir Dar, Ethiopia 0.62 [20], in Northwest Ethiopia 1.61% [10], in Central and Northwest Ethiopia 1.9% [6], a study done in Brazil 1.6% [12]. The discrepancy might be due to study setting, environmental factors, instruments used to diagnose CAs, and time variations. The reason might be due to differences in behaviors of folic acid supplementation before and during pregnancy.

In this study, mothers with low educational status (primary school) were nearly eight times more likely to have congenitally malformed newborns than those with higher levels of education. This finding was consistent with a study conducted in Tanzania [19]. This indicates a potential impact of maternal education on congenital malformation of the newborns. It is widely accepted that maternal factors that are attributed to lower educational status, such as drug use, cigarette smoking during pregnancy, and nutrition, might be responsible for negative neonatal outcomes, including birth defects. In addition to this, the higher occurrence of congenital malformations like central nervous system anomalies could be explained by a lack of foods fortified with folic acid, very low conventional intake of folic acid, and inadequate dietary intake of foods rich in folic acid, such as vegetables due to lack of health information and recommendations as noted in mothers with low educational status.

Women who lived in rural residences were five times more likely to have congenital malformed offspring as compared to those who lived in urban residences. This finding is consistent with the finding in Bishoftu, Ethiopia showing that women who live in urban resident were reduce the occurrence of CAs in their babies by 70% [32]. In contrast, a study revealed that women who live in urban residences were two times more likely to have infants with CAs than women who live in rural residences [20]. The possible reason might be the women who live in rural residences have less chance to be educated, lack the autonomy to make decisions, are less exposed to mass media, and find it challenging to reach health facilities due to the unavailability of healthcare institutions in shorter distances. Women who live in rural areas might have a higher risk of giving birth with congenital defects due to lack of access to quality healthcare services, poor nutrition habits during pregnancy, exposure to environmental pollution, contaminated water sources by pesticides and other toxins in rural residence can harm the developing fetus and lack of information about folic acid supplementation, and all of these factors can lead to congenital malformation in the fetus during critical stages of embryo development of embryogenesis [33].

Women who had a history of CA offspring in the previous gravidity were three times more prone to newborns with CAs reappearance. In this study, there is a significant relationship that was perceived between a previous history of congenital deformities and a higher risk of new CAs. This finding is consistent with a study done in Iraq shown that a maternal history of previous CAs in their offspring was 59 times more likely to reoccurrence CAs in their child as compared to those who had no previous history [34]. This is because women who had a previous history of CA offspring linked to the reoccurrence of chromosomal deformity for future pregnancy are consistent with evidence linking underlying chromosomal or genetic abnormalities to recurrence either live‐born or stillborn [34]. Parents with one child with a neural tube defect have a recurrence rate of 2%–5%, and parents with a history of one child with Down syndrome have the recurrence risk of 1% [34].

This study also showed that women with medical illnesses were seven times more susceptible to newborns with CAs than their counterparts. This finding was congruent with studies conducted in Northwest Ethiopia [35], a study conducted in Southeast Ethiopia [36], and a study done in Erbil City, Iraq [4]. The possible reason might be that comorbidities like pregestational diabetes were identified to be an important risk factor for structural anomalies due to the teratogenic effect of poorly controlled diabetes and is considered as a risk factor during the early period of pregnancy, especially during the first 8 weeks, at which active differentiation of organ systems could occur. Maternal hyperglycemia may result in increased glucose levels in the embryo; consequently, biochemical aberrations increase oxidative stress that leads to cellular apoptosis. Oxidative stress can result in the inhibition of the Pax3 gene especially in the processes of neurulation [37]. Furthermore, oxidative stress is caused by an imbalance between the generation of oxygen free radicals and the cells’ antioxidant defense system, which can result in irreversible DNA oxidation, enzyme inactivation, and congenital deformity that causes death [36].

The present study also showed that there was a strong association between lack of intake of folic acid during early pregnancy and the occurrence of CAs. Mothers of newborns who did not get folic acid supplementation during their early pregnancy were three times more likely to have newborns with CAs, most of which were neural tube defects. This was congruent with studies in Addis Ababa and the Amhara Region, Ethiopia [1]; Northwest Ethiopia [10]; Oromia, Ethiopia [32]; East and West Gojjam zones, Northwest Ethiopia [35]; Mwanza, Tanzania [19]. The possible reason might be a higher occurrence of CAs could be described by a lack of foods fortified with folic acid, due to physiologic changes of pregnancy like poor appetite, nausea, and vomiting results; very low conventional intake of folic acid, loss of folic acid and insufficient nutritional intake of foods rich in folic acid, such as vegetables and animal meat like liver. Folic acid is vital during times of fast growth in infancy as it plays a role in DNA and RNA synthesis which is the body's genetic material [38]. A common mutation in the methylenetetrahydrofolate reductase gene has been identified during folic acid deficiency which produces a thermolabile variant of methylenetetrahydrofolate reductase with reduced enzyme activity, which poses a risk for CA [39]. Daily maternal intake of folic acid alone or in the form of multivitamin supplements before conception until the first trimester of pregnancy can help prevent the occurrence and recurrence of CAs, especially neural tube defects [36].

Similarly in this study, newborns of mothers who had a history of abortion were three times more prone to have newborns with CAs than counterparts. This finding was supported by studies conducted in Denmark [40]. The possible reason might be that a history of spontaneous abortion in newborn mothers has been related to an increased risk of CAs in the next offspring, and spontaneous abortion and CAs share common genetic predisposing factors. The current studies recommended that genetic variants related to the synthesis pathway of nicotinamide adenine dinucleotide, a biological molecule that contributes to many metabolic reactions, result in congenital malformation and spontaneous abortion in humans [40].

Women who had exposure to pesticides were nine times more disposed to have newborns with CAs. This finding was consistent with studies conducted in Southeast Ethiopia that revealed that pesticide exposure during pregnancy five times more prone to congenital deformity [36], a study conducted in Addis Ababa and Amhara Region, Ethiopia [1], and an unmatched case–control study done in Southeast Ethiopia [41]. The possible reason might be chemical exposure during early pregnancy like insecticides, pesticides, fungicides, and other chemicals can cross placenta barriers and affect the developing fetus resulting in CAs. Pesticides accumulate in the placenta that raise and cross the placenta barriers to expose the fetus resulting in health risks and congenital deformity especially neural tube defects and congenital heart defects. The risk of CA increases significantly during early pregnancy due to exposure to pesticides that can disrupt fetal development by the mechanism of interfering with gene expression, cell division, and hormonal balance, actually resulting in malformations in developing organs [42].

## 5 Conclusion and Recommendation

The study's results showed that, in comparison to earlier works of literature, the study area had a high burden of congenital abnormalities (2.7%). Rural residence, history of CA, medical illness, history of abortion, and exposure to pesticides were factors significantly associated with CAs, whereas higher levels of education and folic acid consumption before and during pregnancy were protective factors. Strengthening antenatal care services, promoting periconceptional folic acid supplementation, and minimizing maternal exposure to environmental risk factors may reduce the prevalence. Further studies are recommended to explore genetic and environmental interactions contributing to these anomalies.

## Author Contributions

Daniel Tsega conceived the study and wrote the original draft of the manuscript, analyzed data and its interpretation, participated in the revision, conceptualization, investigation, methodology, and validation the manuscript. Daniel Tsega and Atsede Getawey revised and edited the manuscript, data curation, supported data collection, and revised the manuscript. All authors read and approved the final version of the manuscript.

## Funding

The authors have nothing to report.

## Ethics Statement

Ethical clearance was obtained from the Institutional Review Board (Health Research Ethics Review Committee) of College of Health Science of Wolkite University, and then the formal letter was written by the College of Health Sciences to the concerned office and then submitted to the Gurage, Kembata, and Halaba zone public hospital administrative office before beginning the data collection. Permission was taken from each hospital. This approach aligns with national and local ethical guidelines governing multicenter research. The National Research Ethics Review Guideline (2014) emphasizes that all research protocols, including retrospective studies, must undergo ethical review to ensure the protection of participants’ rights and confidentiality. Specifically, the guideline mandates that researchers obtain approval from an Institutional Review Board before commencing their study. Our research requires that for minimal‐risk studies as it was retrospective chart reviews study, a single Institutional Review Board approval may be sufficient, provided that institutional permissions from participating centers were obtained. Data were taken from charts of the client's records. Therefore, the confidentiality of the data was kept, and the data were not used for other purposes other than the purpose of the current study.

## Consent

Patient consent was not taken for this chart review study, as our study involved a retrospective chart review with no direct patient contact, and that data confidentiality was strictly maintained.

## Conflicts of Interest

The authors declare no conflicts of interest.

## Supporting information




Supplementary File 1: puh270154‐sup‐0001‐Questionaire.docx

## Data Availability

The data set used or analyzed during this study is available from the corresponding author upon reasonable request.

## References

[1] M. Taye , M. Afework , W. Fantaye , E. Diro , and A. Worku , “Factors Associated With Congenital Anomalies in Addis Ababa and the Amhara Region, Ethiopia: A Case‐Control Study,” BMC Pediatrics 18, no. 1 (2018): 142. 29699508 10.1186/s12887-018-1096-9 PMC5921791

[2] “Birth Defect Surveillance Manual Toolkit,” CDC, published 2020, https://www.cdc.gov.

[3] C. Serra‐Juhe , B. Rodríguez‐Santiago , I. Cuscó , et al., “Contribution of Rare Copy Number Variants to Isolated Human Malformations,” PLoS ONE 7, no. 10 (2012): e45530. 23056206 10.1371/journal.pone.0045530 PMC3463597

[4] D. Çanaku , E. Toçi , E. Roshi , and G. Burazeri , “Prevalence and Factors Associated With Congenital Malformations in Tirana, Albania, During 2011–2013,” Materia Socio‐Medica 26, no. 3 (2014): 158–162. 25126007 10.5455/msm.2014.26.158-162 PMC4130685

[5] G. Musumeci , P. Castrogiovanni , F. M. Trovato , R. Parenti , M. A. Szychlinska , and R. Imbesi , “Pregnancy, Embryo‐Fetal Development and Nutrition: Physiology Around Fetal Programming,” Journal of Histology and Histopathology 2, no. 1 (2015): 1–12. http://www.hoajonline.com/journals/pdf/2055‐7091X‐7242‐7241.pdf, 10.7243/2055-7091X-7242-7241.

[6] M. Taye , M. Afework , W. Fantaye , E. Diro , and A. Worku , “Magnitude of Birth Defects in Central and Northwest Ethiopia From 2010–2014: A Descriptive Retrospective Study,” PLoS ONE 11, no. 10 (2016): e0161998. 27706169 10.1371/journal.pone.0161998 PMC5051902

[7] S. Vatankhah , M. Jalilvand , S. Sarkhosh , M. Azarmi , and M. Mohseni , “Prevalence of Congenital Anomalies in Iran: A Review Article,” Iranian Journal of Public Health 46, no. 6 (2017): 733–743. 28828315 PMC5558066

[8] A. Christianson , C. P. Howson , and B. Modell , Global Report on Birth Defect. The Hidden Toll of Dying and Disabled Children (New York, 2006).

[9] A. R. Mohammed , S. A.‐R. Mohammed , and A. M. H. AbdulFatah , “Congenital Anomalies Among Children: Knowledge and Attitude of Egyptian and Saudi Mothers,” Journal of Biology, Agriculture and Healthcare 3, no. 20 (2013): 18–29.

[10] G. Seyoum and F. Adane , “Prevalence and Associated Factors of Birth Defects Among Newborns at Referral Hospitals in Northwest Ethiopia,” Ethiopian Journal of Health Development 32, no. 3 (2018): 156–162.

[11] J. H. da Silva , A. C. P. Terças , L. C. B. Pinheiro , G. V. A. França , M. Atanaka , and L. Schüler‐Faccini , “Profile of Congenital Anomalies Among Live Births in the Municipality of Tangará da Serra, Mato Grosso, Brazil, 2006–2016,” Epidemiologia e Serviços De Saúde 27 (2018): e2018008. 30365695 10.5123/S1679-49742018000300017

[12] H. W. Cosme , L. S. Lima , and L. G. Barbosa , “Prevalence of Congenital Anomalies and Their Associated Factors in Newborns in the City of São Paulo From 2010 to 2014,” Revista Paulista De Pediatria 35 (2017): 33–38. 28977314 10.1590/1984-0462/;2017;35;1;00002 PMC5417807

[13] C. M. da Silva Costa , S. G. N. da Gama , and M. do Carmo Leal , “Congenital Malformations in Rio de Janeiro, Brazil: Prevalence and Associated Factors,” Cadernos De Saude Publica 22 (2006): 2423–2431. 17091179 10.1590/s0102-311x2006001100016

[14] R. Francine , S. Pascale , and H. Aline , “Congenital Anomalies: Prevalence and Risk Factors,” Mortality 1 (2014): 58–63.

[15] X. Zhang , L. Chen , X. Wang , et al., “Changes in Maternal Age and Prevalence of Congenital Anomalies During the Enactment of China's Universal Two‐Child Policy (2013–2017) in Zhejiang Province, China: An Observational Study,” PLOS Medicine 17, no. 2 (2020): 1003047. 10.1371/journal.pmed.1003047 PMC7039412 32092053

[16] L. M. El‐Attar , A. A. Bahashwan , A. D. Bakhsh , and Y. M. Moshrif , “The Prevalence and Patterns of Chromosome Abnormalities in Newborns With Major Congenital Anomalies: A Retrospective Study From Saudi Arabia,” Intractable & Rare Diseases Research 10, no. 2 (2021): 81–87. 33996352 10.5582/irdr.2021.01016 PMC8122309

[17] A. E. Ajao and I. A. Adeoye , “Prevalence, Risk Factors and Outcome of Congenital Anomalies Among Neonatal Admissions in OGBOMOSO, Nigeria,” BMC Pediatrics 19, no. 1 (2019): 88. 30943931 10.1186/s12887-019-1471-1 PMC6446329

[18] M. A. El Koumi , E. A. Al Banna , and I. Lebda , “Pattern of Congenital Anomalies in Newborn: A Hospital‐Based Study,” Pediatric Reports 5, no. 1 (2013): 20–23. 10.4081/pr.2013.e5 PMC3649744 23667734

[19] F. Mashuda , A. Zuechner , P. L. Chalya , B. R. Kidenya , and M. Manyama , “Pattern and Factors Associated With Congenital Anomalies Among Young Infants Admitted at Bugando Medical Centre, Mwanza, Tanzania,” BMC Research Notes 7, no. 1 (2014): 195. 24679067 10.1186/1756-0500-7-195 PMC3974194

[20] D. Mekonnen , M. Taye , and W. Worku , “Congenital Anomalies Among Newborn Babies in Felege‐Hiwot Comprehensive Specialized Referral Hospital, Bahir Dar, Ethiopia,” Scientific Reports 11, no. 1 (2021): 11027. 34040058 10.1038/s41598-021-90387-0 PMC8154920

[21] M. Taye , M. Afework , W. Fantaye , E. Diro , and A. Worku , “Congenital Anomalies Prevalence in Addis Ababa and the Amhara Region, Ethiopia: A Descriptive Cross‐Sectional Study,” BMC Pediatrics 19, no. 1 (2019): 234. 31296186 10.1186/s12887-019-1596-2 PMC6625051

[22] M. Silesh , T. Lemma , B. Fenta , and T. Biyazin , “Prevalence and Trends of Congenital Anomalies Among Neonates at Jimma Medical Center, Jimma, Ethiopia: A Three‐Year Retrospective Study,” Pediatric Health, Medicine and Therapeutics 12 (2021): 61–67. 33628075 10.2147/PHMT.S293285 PMC7898197

[23] R. Francine , S. Psascale , and H. Aline , “Congenital Anomalies: Prevalence and Risk Factors,” Universal Journal of Public Health 2, no. 2 (2014): 58–63, http://www.hrpub.org, 10.13189/ujph.12014.020204.

[24] A. Alborz , “Environmental Characteristics and Prevalence of Birth Defects Among Children in Post‐War Iraq: Implications for Policies on Rebuilding the Iraqi Education System,” Medicine, Conflict and Survival 29, no. 1 (2013): 26–44, http://www.ncbi.nlm.nih.gov/pubmed/23729096. 23729096 10.1080/13623699.2013.765197

[25] R. Kalaskar , A. Kalaskar , F. S. Naqvi , G. S. Tawani , and D. R. Walke , “Prevalence and Evaluation of Environmental Risk Factors Associated With Cleft Lip and Palate in a Central Indian Population,” Pediatric Dentistry 35, no. 3 (2013): 279–283. 23756316

[26] “Congenital Anomalies—Birth Defects Surveillance Toolkit,” Centers for Disease Control and Prevention, published 2019, https://www.cdc.gov/ncbddd/birthdefects/surveillancemanual/facilitators‐guide/module‐1/mod1‐2.html.

[27] WHO/CDC/ICBDSR , Birth Defects Surveillance: A Manual for Programme Managers (World Health Organization, 2014).

[28] F. S. Nino , Sustainable Development Goals—United Nations (United Nations Sustainable Development, 2015).

[29] L. Liu , S. Oza , D. Hogan , et al., “Global, Regional, and National Causes of Under‐5 Mortality in 2000–15: An Updated Systematic Analysis With Implications for the Sustainable Development Goals,” Lancet 388, no. 10063 (2017): 3027–3035. 10.1016/S0140-6736(16)31593-8 PMC5161777 27839855

[30] S. Sarkar , C. Patra , M. K. Dasgupta , K. Nayek , and P. R. Karmakar , “Prevalence of Congenital Anomalies in Neonates and Associated Risk Factors in a Tertiary Care Hospital in Eastern India,” Journal of Clinical Neonatology 2, no. 3 (2013): 131–134. 24251257 10.4103/2249-4847.119998 PMC3830148

[31] S. M. Hashimi , “Determinants of Congenital Anomalies in Afghanistan” (master's thesis, Royal Tropical Institute (KIT) & Vrije Universiteit Amsterdam, 2017).

[32] S. Gedamu , E. G. Sendo , and W. Daba , “Congenital Anomalies and Associated Factors Among Newborns in Bishoftu General Hospital, Oromia, Ethiopia: A Retrospective Study,” Journal of Environmental and Public Health 2021 (2021): 2426891. 33859704 10.1155/2021/2426891 PMC8026314

[33] N. Moges , D. T. Anley , M. A. Zemene , et al., “Congenital Anomalies and Risk Factors in Africa: A Systematic Review and Meta‐Analysis,” BMJ Paediatrics Open 7, no. 1 (2023): e002022. 37429669 10.1136/bmjpo-2023-002022 PMC10335447

[34] S. K. Ameen , S. K. Alalaf , and N. P. Shabila , “Pattern of Congenital Anomalies at Birth and Their Correlations With Maternal Characteristics in the Maternity Teaching Hospital, Erbil City, Iraq,” BMC Pregnancy and Childbirth 18, no. 1 (2018): 501. 30563491 10.1186/s12884-018-2141-2 PMC6299654

[35] B. Tsehay , D. Shitie , A. Lake , E. Abebaw , A. Taye , and E. Essa , “Determinants and Seasonality of Major Structural Birth Defects Among Newborns Delivered at Primary and Referral Hospital of East and West Gojjam Zones, Northwest Ethiopia 2017–2018: Case–Control Study,” BMC Research Notes 12, no. 1 (2019): 495. 31399144 10.1186/s13104-019-4541-4 PMC6688374

[36] S. Jemal , E. Fentahun , M. Oumer , and A. Muche , “Predictors of Congenital Anomalies Among Newborns in Arsi Zone Public Hospitals, Southeast Ethiopia: A Case‐Control Study,” Italian Journal of Pediatrics 47 (2021): 143. 34193221 10.1186/s13052-021-01093-6 PMC8243734

[37] S. Lin , A. Ren , L. Wang , et al., “Aberrant Methylation of Pax3 Gene and Neural Tube Defects in Association With Exposure to Polycyclic Aromatic Hydrocarbons,” Clinical Epigenetics 11, no. 1 (2019): 13. 30665459 10.1186/s13148-019-0611-7 PMC6341549

[38] N. Moges , E. Sisay Chanie , R. M. Anteneh , et al., “The Effect of Folic Acid Intake on Congenital Anomalies. A Systematic Review and Meta‐Analysis,” Frontiers in Pediatrics 12 (2024): 1386846. 39100647 10.3389/fped.2024.1386846 PMC11294162

[39] D. S. Froese , M. Huemer , T. Suormala , et al., “Mutation Update and Review of Severe Methylenetetrahydrofolate Reductase Deficiency,” Human Mutation 37, no. 5 (2016): 427–438. 26872964 10.1002/humu.22970

[40] H. Ji , H. Liang , Y. Yu , et al., “Association of Maternal History of Spontaneous Abortion and Stillbirth With Risk of Congenital Heart Disease in Offspring of Women With vs Without Type 2 Diabetes,” JAMA Network Open 4, no. 11 (2021): e2133805. 34757411 10.1001/jamanetworkopen.2021.33805 PMC8581719

[41] A. G. Mekonnen , A. G. Hordofa , T. T. Kitila , and A. Sav , “Modifiable Risk Factors of Congenital Malformations in Bale Zone Hospitals, Southeast Ethiopia: An Unmatched Case‐Control Study,” BMC Pregnancy and Childbirth 20, no. 1 (2020): 129. 32106830 10.1186/s12884-020-2827-0 PMC7045613

[42] K. Felisbino , S. D. S. Milhorini , N. Kirsten , K. Bernert , R. Schiessl , and I. C. Guiloski , “Exposure to Pesticides During Pregnancy and the Risk of Neural Tube Defects: A Systematic Review,” Science of the Total Environment 913 (2024): 169317. 38104833 10.1016/j.scitotenv.2023.169317

