# Supplementary material for: Prevalence of Congenital Anomalies and Its Associated Factors Among Newborns in Central Ethiopia Region Public Hospitals, Ethiopia: A Retrospective Cross‐Sectional Study, 2023
Source: Public Health Chall. 2025 Oct 30;4(4):e70154. doi: 10.1002/puh2.70154 (PMC12573466; doi:10.1002/puh2.70154)
Supplement: Supplementary file 1 — puh270154‐sup‐0001‐Questionaire.docx [file PUH2-4-e70154-s001.docx]

**ANNEX II: ENGLISH VERSION PARTICIPANT CONSENT FORM**

The information is read and explained to me the participant information sheet. I have clearly understood the propose of the research, the procedures, the risk and benefits, issues of confidentiality, the rights of participating and the contact address of any queries. I have been given the opportunity to ask questions for the things that may have been unclear. I am informed that I have the right to withdraw from the study at any time or not to answer any questions that I do not want. Therefore, I declare my voluntary consent to participate in this study.

If participant does not agree to be interviewed thanks her and go to the next participant.

If respondent say **YES** continue.

**Data Collector agreement**

“I certify that I have filled the questionnaire in accordance with the training that is given to me and instructions stated in it. I have confirmed that the collected information is correct.”

Name of Data collector: ____________________________Signature _______________

Date**______/_____/________**E.C

Name of hospital_________________

**Checked by:** supervisor for completeness:

Supervisor Name __________________________signature______________

Date______/_____/_______E.C.

Table 3: Questionnaires to assess the magnitude of congenital anomalies and determinant factors among infants in gurage zone public hospitals, southern Ethiopia, 2022.

**Questionnaire English Version**

**Part I**: **socio- demographic characteristics**

**Instruction**: please encircle the number listed before the option to indicate your response and fill the black for without option.

Code of questionnaire _________________ Respondents’ signature _____________

| S/no | Questions | Answers/choices |
| --- | --- | --- |
| 1 | Infants sex | 1. Male 2. Female |
| 2 | Infants age | ___________complete months |
| 3 | Newborn status | 1. Livebirth 2. Stillbirth 3. Medically terminated |
| 4 | Birth order | 1. First 2. Second 3. Third 4. Fourth 5. Fifth and above |
| 5 | Infant mother age | ___________complete years |
| 6 | Infant mother religion | 1. Orthodox 2. Protestant 3. Muslim 4. catholic 5. Others (Specify)________ |
| 7 | Infant mother ethnicity | 1. Kembata 2. Amhara 3. Oromo 4. Halaba 5. Other (specify) _________ |
| 8 | Infant father ethnicity | 1. Kembata 2. Amhara 3. Oromo 4. Halaba 5. Other (specify) _________ |
| 9 | Infants parent Marital status | 1. Single 2. Married 3. Divorced 4. Widowed |
| 10 | Infants mother educational status | 1. No education 2. Primary school 3. Secondary school 4. Occupational training 5. Diploma and above |
| 11 | Infants father educational status | 1. No education 2. Primary school 3. Secondary school 4. Occupational training 5. Diploma and above |
| 12 | Infants mother occupation | 1. House wife 2. Factory worker 3. Government/private employee 4. Merchant 5. Student 6. Other specify ____________ |
| 13 | Infants father occupation | 1. Farmer 2. Government/private employee 3. Merchant 4. Student 5. Daily labor 6. Other specify ____________ |
| 14 | Residence | 1. Rural 2. Urban |

**Part II: Maternal Genetical Factors**

| 15 | Is there any close family member with history of congenital anomalies? | 1. No 2. Yes |
| --- | --- | --- |
| 16 | Do you have history of congenital anomalies affected pregnancy? | 1. No 2. Yes |
| 17 | Do you have genetically relationship with your husband? | 1. No 2. Yes |

**Part III: Maternal reproductive and obstetric history**

| 18 | Number of Gravidity | 1. One 2. Two 3. three 4. Four 5. Five and above |
| --- | --- | --- |
| 19 | Number of parity | 1. One 2. Two-Four 3. Five and above |
| 20 | Do you have any history of Abortion? | 1. No 2. Yes |
| 21 | Did you have history of newborn deaths? | 1. No 2. Yes (Still birth or Early neonatal loss) |
| 22 | If, yes specify the types of death | 1. Still birth 2. Early neonatal loss |
| 23 | How many children’s do you have currently? | 1. One 2. Two 3. Three 4. Four 5. Five and above |
| 24 | Was your pregnancy planed | 1. No 2. Yes |
| 25 | Gestational age at 1st ANC (antenatal care) visit | 1. No ANC visit 2. 1-3 months 3. 4 months and above |
| 26 | How many times did you receive ANC? | 1. One 2. Two 3. Three 4. Four and above |
| 27 | Where did you receive ANC? | 1. Public health institution 0. Yes 1. No 2. Private health institution 0. Yes 1. No |
| 28 | How many times had you got preterm delivery | 1. None 2. One 3. Two and above |
| 29 | Did you had history of Neonate/Infant/child death? | 1. No 2. Yes |
| 30 | If yes, how many Neonate/Infant/child death occurred? | 1. One 2. Two 3. Three and above |

**Part IV: Maternal Medical and Drug History**

| 31 | Have you ever been told by a doctor that you had any of these diseases: Hypertension, Hyper/hypothyroidism, bladder /urinary tract infection, tumor, epilepsy, DM | 1. No 2. Yes |
| --- | --- | --- |
| 32 | If yes did you take any medication (for the medical condition)? | 1. Yes 2. No |
| 33 | If “yes” list the medication name | ________________________________ |
| 34 | Did you used any hormonal contraceptive methods? | 1. No 2. Yes |
| 35 | Have you experienced any fever/ febrile illness? | 1. No 2. Yes |
| 36 | If “yes” did you take any antipyretic (fever reducing medication)? | 1. No 2. Yes |

**Part V: Maternal Chemical Exposure**

| 37 | Is there waste disposal site or industry near to your residence? | 1. No 2. Yes |
| --- | --- | --- |
| 38 | If “yes” state the distance from your house | _________________ meter |
| 39 | Have you used any pesticides (insecticides, herbicides or fungicides) at home /work place? | 1. No 2. Yes |
| 40 | Have you ever used sauna/hot tub? | 1. No 2. Yes |
| 41 | Have you experienced any diagnostic/therapeutic radiations? | 1. No 2. Yes |

**Part VI: Maternal nutritional and folic acid consumption**

| 42 | What was your pre-pregnancy weight and height | Wt. _______ Kg, Ht________ meter |
| --- | --- | --- |
| 43 | Did you take Folic acid supplements? | 1. Never 2. Peri-conceptional intake 3. After 3 months of conception |

**Part VII: maternal lifestyle**

| 43 | Did you take any caffeine | 1. No 2. Yes |
| --- | --- | --- |
| 45 | If your answer is yes which caffeine source | 1. Coffee 2. Tea |
| 46 | How many cups per day (in average)? | ______________ |
| 47 | Did you drink alcohol? | 1. No 2. Yes |
| 48 | If your answer yes (amount in average) | 1. >500ml/day 2. <500ml/day 3. Occasional (<500ml/week) |
| 49 | Did you smoke? | 1. No 2. Yes |
| 50 | Did you had any home/occupational exposure to tobacco smoke? | 1. No 2. Yes |

**Part VII: Magnitude related questions**

| 51 | Is your new-born/infant has any congenital anomalies? | 1. No 2. Yes |
| --- | --- | --- |
| 52 | If yes, which types of congenital anomalies have your infants? | 1. Central nervous system abnormalities 2. Neural tube defect 3. Anencephaly 4. Hydrocephalous, Spinal bifida 5. Gastro-intestine defect 6. Exomphalos 7. Gastroschisis 8. Imperforate anus 9. Respiratory system defect 10. trachea-Esophageal atresia 11. trachea esophageal fistula 12. Orofacial Clefts 13. cleft lip 14. cleft palate 15. cleft lip and cleft palate 16. Genitourinary anomalies 17. Hypospadias 18. Epispadia 19. Musculoskeletal deformities 20. Polydactyl 21. Syndactly 22. Clinodactyly 23. clubfoot 24. Congenital cardiac defects 25. Atrial septal defect 26. Ventricular septal defect 27. Chromosomal abnormalities (down syndrome) 28. Others specify ________________ |
